# Prophylactic IVC filter placement in bariatric surgery patients: results from a prospective filter registry

**DOI:** 10.1186/s42155-018-0021-5

**Published:** 2018-11-15

**Authors:** Alexander Y. Sheu, Nam Sao Hoang, Andrew J. Kesselman, Tie Liang, Jarrett K. Rosenberg, William T. Kuo

**Affiliations:** Division of Vascular and Interventional Radiology, Stanford University Medical Center, 300 Pasteur Drive, H-3651, Stanford, CA 94305-5642 USA

**Keywords:** Inferior vena cava filter, Acute PE, Bariatric surgery

## Abstract

**Background:**

Bariatric surgery patients are at increased risk for VTE, but potential risks versus benefits of IVC filters in this group remain unclear. Indwelling filters may increase risk of VTE, and removal of filters in obese patients can be challenging. This study evaluated the incidence of VTE in select bariatric patients receiving prophylactic IVC filters, their risk of filter-related complications, and outcomes from attempted filter retrieval.

**Results:**

Postsurgical DVT occurred in 3 patients within 3 months postoperatively (3%)(95%CI:1–9%), and 1 patient(1%)(95%CI:0–5%) developed acute low-risk PE at 31 days postoperatively, prior to filter removal. All VTE patients were successfully managed with therapeutic anticoagulation alone except one who required thrombolysis. Median filter dwell time was 54 days (range:22–1548), and there were no major filter-related complications (0%)(95%CI:0–3%). Retrieval was attempted in 104 cases (97%)(95%CI:92–99%) and successful in 104 cases (100%)(95%CI:97–100%). Thirty-three patients (32%)(95%CI:23–42%) required advanced techniques for filter removal, and there were no major procedural complications (0%)(95%CI:0–3%). Median follow-up occurred at 344 days (range:3–1570) days after filter retrieval.

**Conclusions:**

No cases of life-threatening post-op PE occurred in this cohort of high-risk bariatric surgery patients receiving prophylactic IVC filters in combination with mechanical and chemoprophylaxis. The risk of filter-related complications was low and retrieval success was high with adjunctive use of advanced techniques.

**Clinical trial registration:**

NCT01158482

## Background

Patients undergoing bariatric surgery such as Roux-en-Y gastric bypass (RYGB) and sleeve gastrectomy (SG) are at increased risk for venous thromboembolism (VTE) (ASMBS 2013), but the potential risks versus benefits of inferior vena cava (IVC) filter placement in this group remain unclear (Rajasekhar and Crowther 2010). The American Society for Metabolic and Bariatric Surgery (ASMBS 2013) states that prophylactic IVC filter placement may be considered in selected patients undergoing bariatric surgery who are felt to be at high risk for venous thromboembolism (ASMBS 2013); however, the optimal protocol for filter management is still unknown, and the potential risks versus benefits of prophylactic filters in this high-risk population remain poorly understood. Indwelling IVC filters may increase the risk of VTE over time, but follow-up and successful removal of IVC filters in obese patients can be challenging (Birkmeyer et al. 2013). The purpose of this study was to evaluate the incidence of VTE in bariatric patients receiving prophylactic IVC filters, their risk of filter-related complications, and the outcomes from attempted IVC filter retrieval.

Over 4 years, 107 high-risk patients receiving a retrievable IVC filter prior to bariatric surgery were prospectively enrolled in a single-center IRB-approved study. Filters were placed if patients had BMI > 50 kg/m^2^ and/or VTE risk factors per ASMBS guidelines, and all received mechanical (SCDs) and chemo-prophylaxis (UFH/LMWH). All were contacted for postoperative follow-up, and filter retrieval was attempted when no longer needed.

## Methods

### Study design

This study was conducted after institutional review board approval. Over a 4 year period (2013–2016), 766 patients underwent bariatric surgery. From this group, 109 high-risk patients underwent prophylactic IVC filter placement prior to bariatric surgery, and 107 were enrolled in a prospective single-center study (NCT01158482)(2 patients declined). All data were collected electronically using the REDCap system (Nashville, TN), and enrollment was capped at 107 patients for this analysis.

### Study population

All patients undergoing bariatric surgery (laparoscopic RYGB or laparoscopic SG) who were deemed to be at high risk for VTE were included. Specifically, prophylactic IVC filters were placed after informed consent in patients with BMI > 50 kg/m^2^ and/or other major VTE risk factors (Table 1) per ASMBS guidelines (ASMBS 2013) and none refused the filter. One hundred seven patients including 30 men and 77 women with mean age 49 years (range: 25–70 years) undergoing prophylactic IVC filter placement prior to bariatric surgery were included in the study. The mean BMI was 57.8 ± 9.2 kg/m^2^, consistent with Grade 3 (extreme) obesity (Sturm and Hattori 2013). Baseline demographics and clinical characteristics are summarized in Table 1. In addition, all patients received mechanical (SCDs) and chemoprophylaxis (heparin or enoxaparin) in the perioperative setting per standard bariatric surgery protocol. Ninety patients underwent laparoscopic RYGB, and 17 patients underwent laparoscopic SG; perioperative details are summarized in Table 2. Post-surgery, all patients received either heparin (5000 U SQ every 8 h or 30 mg enoxaparin SQ daily) until ambulatory or discharge. Patients on prior anticoagulation were transitioned back to their baseline regimen. Patients with newly diagnosed VTE were initiated on therapeutic anticoagulation for at least 3–6 months. After discharge, in addition to routine follow-up in bariatric surgery clinic, all patients were contacted for filter follow-up by a dedicated interventional radiology IVC filter clinic (comprised of trained clinical coordinators, nurse practitioners, and a supervising IR physician) within 1 to 2 months after IVC filter placement to schedule filter removal. If there was no response, patients were contacted again at 2 to 3-month intervals until successful contact was made and/or a decision was made to postpone filter removal for medical reasons. Following this process, IVC filter retrieval was attempted when filtration was no longer indicated.

**Table 1:** Patient Characteristics

Demographic	Value
Number of patients	107
Age (years)	49 ± 11
BMI (kg/m^2^)	57.8 ± 9.2
Female	77 (72.0)
Race	
White	54 (50.5)
More Than One Race	19 (17.8)
Unknown/Not Reported (includes Hispanic or Latino)	16 (15.0)
Black or African American	15 (14.0)
Native Hawaiian or Other Pacific Island	2 (1.9)
American Indian/Alaska Native	1 (0.9)
Smoking	9 (8.4)
Comorbidities & VTE Risk Factors	
Obstructive sleep apnea	67 (62.2)
Mobility limitations	34 (31.8)
Asthma	24 (22.4)
Prior VTE	24 (22.4)
Chronic venous insufficiency	13 (12.2)
Peripheral vascular disease	3 (2.8)
Thrombophilia	3 (2.8)
Home oxygen	1 (0.9)
Home medications	
Aspirin	12 (11.2)
Warfarin	8 (7.5)
Other oral anticoagulant	3 (2.8)
Enoxaparin	0 (0.0)

Data are presented as mean ± standard deviation or number (%) unless otherwise indicated

**Table 2:** Perioperative details

Perioperative details	Value
Operation	
Roux-en-Y gastric bypass	90 (84.1)
Sleeve gastrectomy	17 (15.9)
Intraoperative VTE prophylaxis	
Subcutaneous heparin	107 (100.0)
Subcutaneous enoxaparin	0 (0.0)
Sequential compression devices	107 (100.0)
Postoperative VTE prophylaxis	
Subcutaneous heparin	105 (98.1)
Subcutaneous enoxaparin	2 (1.87)
Sequential compression devices	107 (100.0)
Days spent inpatient prior to discharge	2 ± 1

Data are presented as mean ± standard deviation or number (%) unless otherwise indicated

### IVC filter placement and retrieval

All patients underwent placement of a retrievable-type IVC filter. Percutaneous transjugular venous access was obtained, and all filters were implanted within the infrarenal IVC under fluoroscopic guidance. During attempted removal, venography was performed to assess for caval thrombosis. In addition, radiographic assessment for filter-related complications was also performed including filter migration, penetration, fracture, and component embolization. Filter retrieval was initially attempted using standard snare technique. If routine retrieval attempts were unsuccessful due to a tip-embedded and/or leg embedded filter, advanced retrieval techniques were attempted using wire loop (Kuo et al. 2006), rigid forceps (RF) (Stavropoulos et al. 2008), laser sheath (Kuo et al. 2013), or combinations of the above. Procedural complications were defined according to established guidelines (Sacks et al. 2003), and major complications were specifically defined as follows: death, permanent adverse sequelae, requirement of major therapy, unplanned increase in level of care, or prolonged hospitalization (> 48 h). Patients on prior anticoagulation were maintained on their baseline regimen without cessation during filter placement and retrieval.

### Clinical outcomes

Primary outcomes were acute deep vein thrombosis (DVT) and/or pulmonary embolism (PE) as assessed by ultrasound (for DVT) and computed tomography (for PE). Acute PE were classified as low risk if there was absence of hemodynamic instability or right ventricular dysfunction (assessed by chest CT and/or echocardiogram), requiring no treatment escalation beyond therapeutic anticoagulation (Tapson 2008).

### Statistics

Data were reported with 95% confidence intervals unless otherwise indicated; the proportion and 95% confidence interval were calculated for each of the following variables by assuming a binomial distribution of each proportion: IVC filter retrieval, successful filter retrieval, filter retrieval complications, filter-related complications, and VTE. The relationship between VTE and each of the continuous VTE risk factors (age, BMI, and length of postoperative hospitalization) were investigated by exact Mann-Whitney ranks test. The relationship between VTE and each of the binary risk factors (preoperative warfarin use, mobility limitations, asthma, home oxygen use, obstructive sleep apnea (OSA), peripheral vascular disease, prior VTE, thrombophilia, chronic venous insufficiency (CVI), preoperative aspirin use, and other preoperative anticoagulant use) were examined by Fisher’s exact test. All statistics and confidence intervals were calculated using Stata Release 14.1 software (StataCorp LLC, College Station, TX).

## Results

### Inferior vena cava filter characteristics

IVC filter placement was successful in 100% (107) of patients with no insertion-related complications (0%). All implanted IVC filters were retrievable, and 95 (88.8%) were Günther Tulip filters (Cook Medical, Bloomington, IN). All filter types are summarized in Table 3. Per protocol, all patients were contacted for IVC filter retrieval after recovery from bariatric surgery. One bariatric patient underwent filter placement and failed retrieval outside our department and was subsequently referred to our filter clinic for retrieval. IVC filter retrieval was attempted in 104 cases (97%)(95%CI:92–99%) and successful in 104 cases (100%)(95%CI:97–100%). IVC filter retrieval was pending at the time of analysis for 1 patient, and 2 patients were noncompliant with follow-up. Thirty-three patients (32%)(95%CI:23–42%) required advanced techniques for IVC filter retrieval. No major complications occurred at the time of IVC filter retrieval (0%)(95%CI:0–3%). There were 4 (4%)(95%CI:1–9%) minor complications: 1 right neck hematoma and 3 IVC pseudoaneurysms that resolved after temporary inflation of a compliant tamponade balloon.

**Table 3:** Filter types

Filter type	Value (%)
Günther Tulip (Cook Medical)	95 (88.8)
Option (Argon Medical Devices)	5 (4.7)
Denali (BARD Peripheral Vascular)	4 (3.7)
ALN (ALN Implants Chirurgicaux)	2 (1.9)
Celect (Cook Medical)	1 (0.9)

**Fig. 1:**
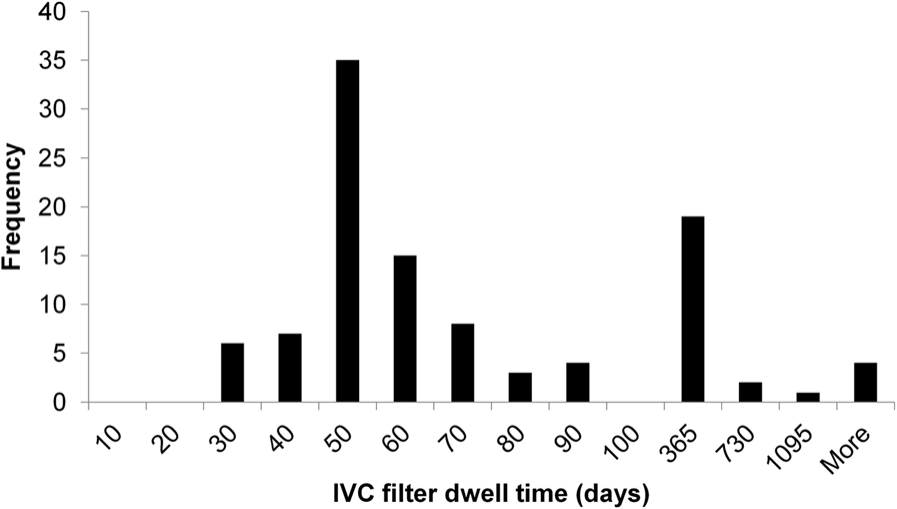
Histogram of IVC filter dwell times. Most filters were retrieved within 90 days. Some filters were retrieved after 1 year, most frequently due to delayed bariatric surgery or noncompliance with initial follow-up requests

Median IVC filter dwell time was 54 (range: 22–1548) days. IVC filter dwell times are summarized in Fig. 1. 73% of IVC filters (*n* = 78) were retrieved within 3 months. The remaining 26 patients had delayed filter removals beyond this window for the following reasons: initial noncompliance with follow-up (*n* = 19); delayed bariatric surgery if the surgeon required additional preoperative weight loss (*n* = 5); medical necessity in patient with DVT (*n* = 1); and failed retrieval attempt outside our department (*n* = 1). All patients with delayed retrievals underwent successful filter removal in our department. There were no major IVC filter-related complications (0%)(95%CI:0–3%). One patient (1%)(95%CI:0–5%) experienced a minor complication reporting pain from a tilted IVC filter (dwell time 85 days) presumed to be filter-related, and this pain resolved after filter retrieval.

### Outcomes

**Fig. 2:**
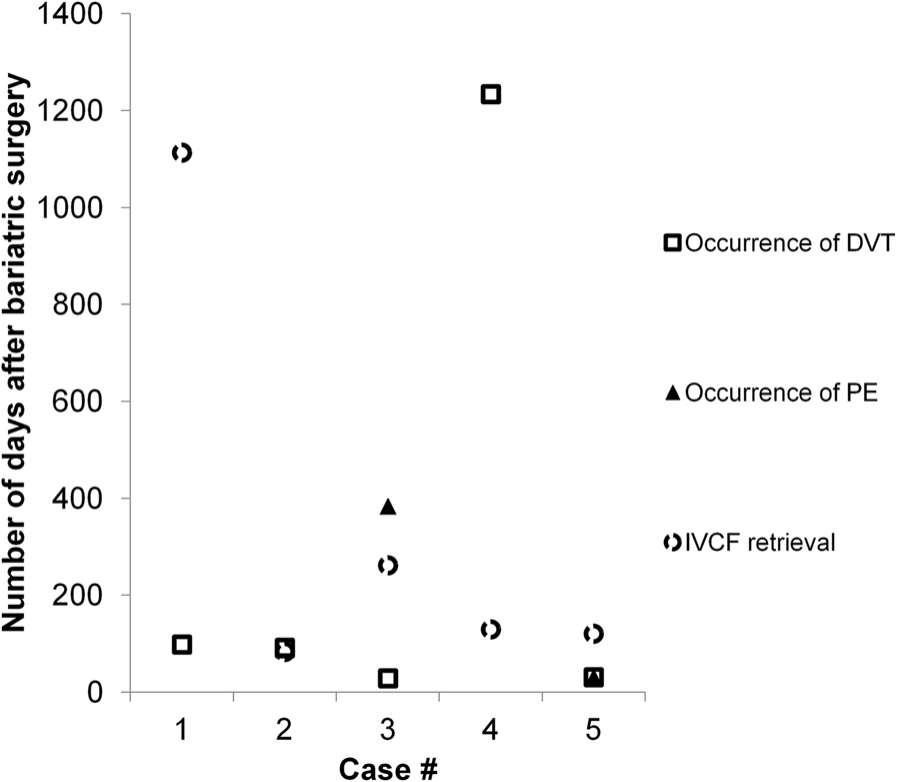
VTE episodes in 5 patients after bariatric surgery. Squares indicate occurrence of DVT; triangles indicate occurrence of PE; circles indicate IVC filter retrieval. VTE events occurring > 3 months postoperatively in cases #3 and #4 were deemed unrelated to bariatric surgery

Patients were routinely followed after IVC filter placement, after bariatric surgery, and after IVC filter retrieval. Following filter removal, clinical follow-up occurred at a median of 344 (range: 3–1570) days after IVC filter retrieval. There were no deaths from VTE (0%)(95%CI:0–3%) and no postoperative mortality (0%)(95%CI:0–3%). Postsurgical DVT occurred in 5 patients (5%)(95%CI:2–11%), and 3 cases (3%)(95%CI:1–9%) (occurring within 3 months postoperatively) were deemed to be related to surgery as shown in Fig. 2. Three patients experienced DVT at 29, 31, and 98 days after bariatric surgery (prior to IVC filter retrieval), and two patients experienced DVT after IVC filter retrieval at 91 and 1234 days after bariatric surgery. The case of acute DVT diagnosed 1234 days postoperatively was classified as unrelated to surgery. Two patients (2%)(95%CI:0–7%) also developed acute low-risk PE at 384 (DVT at 29 days) and 31 (DVT at 31 days) days postoperatively. The acute PE diagnosed 384 days postoperatively was classified as unrelated to surgery. Three VTE patients were successfully managed with therapeutic anticoagulation alone and two required additional catheter directed thrombolysis. One patient was successfully treated with catheter directed thrombolysis for symptomatic acute DVT in the IVC and bilateral iliofemoral veins in the setting of heparin induced thrombocytopenia 29 days postoperatively; acute clot was found to be trapped below the IVC filter and there was no evidence of acute PE. The IVC filter was placed 32 days prior to the DVT, and after the patient recovered and filtration was no longer needed, the filter was successfully retrieved 202 days after bariatric surgery. A second patient was successfully treated with catheter directed thrombolysis for symptomatic IVC and left common iliac DVT occurring 14 days after complex IVC filter retrieval. This event occurred 91 days postoperatively and 98 days after IVC filter placement.

### Risk factors for VTE

In this cohort, the presence of preoperative warfarin use was the only significant risk factor for postoperative VTE (*p* = 0.044). Age (*p* = 0.71), BMI (*p* = 0.79), length of postoperative hospitalization (*p* = 0.93), mobility limitations (*p* = 1.00), asthma (*p* = 0.31), home oxygen use (*p* = 1.00), obstructive sleep apnea (OSA) (*p* = 0.16), peripheral vascular disease (*p* = 1.00), prior VTE (*p* = 0.07), thrombophilia (*p* = 0.14), CVI (*p* = 1.00), preoperative aspirin use (0.46), and other preoperative anticoagulant use (*p* = 1.00) were not found to be significant risk factors for postoperative VTE.

## Discussion

Bariatric surgery patients are at increased risk of VTE compared to the general population due to a combination of factors including underlying morbid obesity, related comorbid conditions, and general immobility (Anderson and Spencer 2003; Allman-Farinelli 2011). Consequently, acute PE is a leading cause of perioperative death in the setting of bariatric surgery accounting for approximately 40% of all deaths (Omalu et al. 2007; Morino et al. 2007; Birkmeyer et al. 2010), and current surgical guidelines recommend routine VTE prophylaxis including chemoprophylaxis with heparin and mechanical prophylaxis with SCDs in patients undergoing bariatric surgery (ASMBS 2013; SAGES 2007). Although the addition of a prophylactic IVC filter has been used in certain patients undergoing bariatric surgery, routine filter placement is not currently recommended due to conflicting evidence regarding the safety and efficacy of IVC filters (Rajasekhar and Crowther 2010). Some studies report decreased incidence of PE with prophylactic IVC filters in “selected patients at high risk of VTE” based on risk factors such as thrombophilia or prior history of VTE (Obeid et al. 2007; Overby et al. 2009). Other studies report no reduction in rates of postoperative VTE or even increased rates of subsequent VTE and IVC filter-related complications, stating that half of the complications resulting in death or permanent disability among IVC filter patients were directly related to the device itself (Birkmeyer et al. 2013; Birkmeyer et al. 2010).

Currently, the most widely accepted indication for IVC filter placement is in patients with proven VTE and absolute or relative contraindications to anticoagulation (Kearon et al. 2016), and the use of prophylactic IVC filters is still under debate. The ASMBS currently states that prophylactic IVC filter placement may be considered in combination with chemical and mechanical prophylaxis for selected high risk patients in whom the risks of VTE are determined to be greater than risks of IVC filter related complications (ASMBS 2013). These guidelines suggest that the addition of a prophylactic IVC filter may help prevent life-threatening postoperative PE in select bariatric surgery patients with severe obesity. Using these guidelines, the mean BMI of patients receiving a prophylactic IVC filter was 57.8 kg/m^2^, consistent with extreme morbid obesity (Ogden et al. 2014). It has previously been observed that IVC filter placement and retrieval can be technically challenging in bariatric surgery patients, requiring specific expertise (Ferrell et al. 2004; Vaziri et al. 2009). In our cohort, 100% of IVC filter placement attempts were successful and uneventful, but prompt and effective filter retrieval required substantial efforts following bariatric surgery. Postoperatively, patients were routinely contacted for follow-up by a dedicated IVC filter clinic. Using this system, the vast majority of patients returned within 3 months for filter removal. We found the overall success rate for retrieval was 100%, but 32% of patients required advanced retrieval techniques to achieve successful filter removal. There were no major filter-related complications at the time of IVC filter retrieval, and these results contrast significantly with studies stating that IVC filters account for half of complications leading to death or permanent disability among bariatric surgery patients receiving prophylactic filters (Birkmeyer et al. 2010). The difference in outcomes may be attributed to heterogeneity in IVC filter types and poor long-term follow-up in the prior study (Birkmeyer et al. 2010), as 46% of IVC filters were permanent (22% unknown types) and the majority were never retrieved (Birkmeyer et al. 2010), conferring the risk of morbidity associated with chronic indwelling filters. Although prior studies have suggested that IVC filters may increase the risk of VTE in bariatric surgery patients (Birkmeyer et al. 2013; Birkmeyer et al. 2010), we believe the long-term risks from indwelling IVC filters can be mitigated with successful follow-up and retrieval. In the current study, no major filter-related morbidity was observed, and 97% of implanted filters were removed.

In 2010, the United States Food and Drug Administration (FDA) issued a Safety Alert for IVC filters recommending that retrievable filters be removed as soon as protection from PE is no longer needed in order to avoid complications from indwelling filters (FDA 2010). In 2014, a follow-up Safety Communication from the FDA reported that the risk-to-benefit ratio favors IVC filter removal within 29 to 54 days after implantation if patient’s risk for PE had passed (FDA 2014). Consistent with these guidelines, the median IVC filter dwell time in our cohort was 54 days; however, it should be noted that some patients with post-op DVT required longer protection from PE so the decision must be individualized. Historically, the reported rates of DVT and PE in bariatric surgery patients are 1 to 3% and 0.3 to 2%, respectively (Rajasekhar and Crowther 2010). In this study, there were three cases (3%) of postoperative DVT deemed to be related to bariatric surgery. Since there was no mortality from any postoperative VTE in our study, including a patient with extensive IVC thrombus burden below the filter (which we believe may have prevented massive PE), it is possible that prophylactic IVC filters provided added protection that could have prevented life-threatening acute PE.

Our study has important limitations. The small number of VTE events would limit the ability to detect and model significant risk factors for VTE. Nevertheless, our results are consistent with a prior study showing that risk factors for postoperative VTE such as CVI, BMI, and OSA did not significantly increase the risk of VTE in bariatric surgery patients (Sapala et al. 2003). Most patients in our study received a single IVC filter type, so the results may not be generalizable to other filters. The high rates of patient follow-up and IVC filter retrieval success may be specific to a specialized center dedicated to managing IVC filters, but perhaps this model could be adopted by other centers. Finally, this is a nonrandomized study not designed to show if an IVC filter can definitively prevent death from postoperative VTE; however, conducting a randomized controlled trial may be ethically challenging in a population at high risk for developing life-threatening PE.

## Conclusion

In conclusion, no cases of life-threatening post-op PE occurred in this cohort of select high-risk bariatric surgery patients receiving prophylactic IVC filters in combination with mechanical and chemoprophylaxis. If ethically feasible, a future randomized trial would be helpful to elucidate these findings especially among other specific groups of bariatric patients at risk for VTE. In our study, the risk of filter-related complications was low when patients were closely followed by a dedicated IVC filter clinic, compliant with prompt return, and underwent a successful retrieval procedure. A high retrieval rate can eliminate potential risks associated with an indwelling filter, but successful filter removal may also depend on advanced retrieval techniques in a specialized center. These results may be used to inform future guidelines and future studies on IVC filter use and acute PE prevention in high-risk bariatric surgery patients.

## Abbreviation

ASMBS: American Society for Metabolic and Bariatric Surgery
CVI: Chronic venous insufficiency
DVT: Deep vein thrombosis
FDA: United States Food and Drug Administration
IR: Interventional radiology
IVC: Inferior vena cava
OSA: Obstructive sleep apnea
PE: Pulmonary embolism
RYGB: Roux-en-Y gastric bypass
SCDs: Sequential compression devices
SG: Sleeve gastrectomy
VTE: Venous thromboembolism
